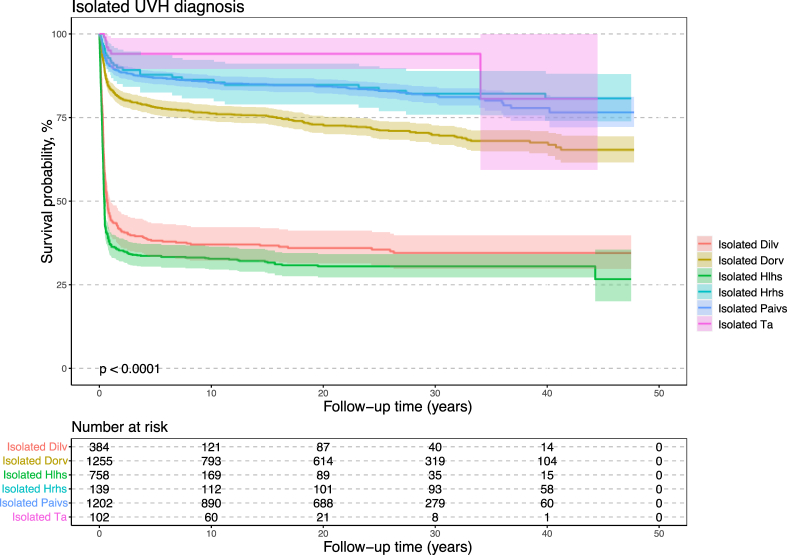# Long-term survival in patients with univentricular heart: A nationwide, register-based cohort study^☆^

**DOI:** 10.1016/j.ijcchd.2024.100503

**Published:** 2024-02-18

**Authors:** Ayse-Gül Öztürk, Mikael Dellborg, Anna Damlin, Kok Wai Giang, Zacharias Mandalenakis, Peder Sörensson

**Affiliations:** a Department of Medicine Solna, Karolinska Institutet, Stockholm, Sweden; b Department of Cardiology, Karolinska University Hospital, Stockholm, Sweden; c Institute of Medicine, Sahlgrenska Academy, University of Gothenburg, Gothenburg, Sweden; d Department of Molecular Medicine and Surgery, Karolinska Institutet, Stockholm, Sweden; e Department of Clinical Physiology, Karolinska University Hospital, Stockholm, Sweden; f Department of Medicine, Geriatrics and Emergency Medicine, Sahlgrenska University Hospital, Gothenburg, Sweden; g Adult Congenital Heart Unit, Department of Medicine, Sahlgrenska University Hospital, Gothenburg, Sweden

**Keywords:** Univentricular heart, Nationwide, Registry study, Survival

## Abstract

**Background:**

Children with univentricular heart (UVH) have a limited life expectancy without early treatment. Long-term survival in UVH, in an unselected nationwide cohort, is unclear.

**Objectives:**

To determine long-term survival in patients with UVH including non-operated patients compared with a control population in Sweden.

**Methods:**

Patients with UVH born between 1970 and 2017 were identified from the National Registers and were matched for birth year and sex with 10 individuals without congenital heart disease. Follow-up was from birth until death, transplantation, or the end of study. Mortality risk was estimated by Cox proportional regression models and Kaplan–Meier survival analysis.

**Results:**

We included 5075 patients with UVH including 758 (14.9%) patients with hypoplastic left heart syndrome (HLHS), and 50,620 matched controls. Median follow-up time was 13.6 (IQR 0.7; 26.8) years. The hazard ratio for death in patients with UVH was 53.0 (95% confidence interval, 48.0–58.6), and for HLHS, 163.5 (95% CI, 124.3–215.2). In patients with HLHS, 84% of those who were born between 1982 and 1993 died or had transplantation during the first year of life compared with 29% born between 2006 and 2017. In patients with UVH without HLHS, death/transplantation in the first year of life declined from 36% in those born between 1970 and 1981 to 8.7% in those born between 2006 and 2017.

**Conclusions:**

The risk of mortality was >50 times higher in patients with UVH than in controls. The survival rate increased with a later decade of birth but was still <75% in patients born with HLHS.

## 1 Introduction

In recent decades, surgical and medical improvements have led to increased survival in patients with congenital heart defects [1]. This improvement in survival has led to the development of a large and growing population of adults with congenital heart disease. The adult congenital heart disease population now numerically exceeds the pediatric congenital heart population [2]. One of the most complex congenital heart defects is univentricular heart (UVH). UVH is a broad term that covers various cardiac structural abnormalities in which one ventricle is severely underdeveloped or a ventricular septal wall does not form. If patients with UVH remain untreated, they have a limited life expectancy [3,4] and a generally poor prognosis compared with patients with other congenital heart defects [5]. Simultaneously, there has been a shift in ACHD care towards a focus on long-term management, including arrhythmias, cardiovascular complications, heart failure [[5], [6], [7], [8], [9], [10], [11]] and Fontan-associated liver disease [12].

There were no treatment options for neonates with hypoplastic left heart syndrome (HLHS) in the 1970s. Hence, mortality was very high within the first week of life [4]. The UVH requires numerous surgical procedures, eventually leading to the Fontan-type of circulation [13]. The Fontan procedure was developed in 1971 to divert systemic venous return to the pulmonary artery [14] and has undergone multiple modifications [15,16]. From the first performed Fontan procedures in 1975–1977, when the mortality rate was 30% [17], the results have improved significantly. In 1975, Elliott et al. reported the use of prostaglandin E1 infusion in 2 children with cyanotic congenital heart disease [18]. This infusion raises oxygen saturation as an effect of preserving the patency of the ductus arteriosus [19]. This treatment has become an essential part of the therapy. In Sweden, the first Norwood procedure in a patient with HLHS was performed in 1993.

While short-term outcomes in children with UVH have been studied [3,20], results showing the long-term effects in the UVH population after childhood, particularly from a nationwide cohort, are still lacking. We studied an entire nation over a long period of time and included all patients who were born with UVH, even patients who were not surgically treated.

## 2 Methods

### 2.1 Aim

The primary aim of our study was to examine the survival trends in patients with UVH from birth until the age of 47 years. The secondary aim was to evaluate the risk of mortality in patients with UVH compared with matched controls without congenital heart disease.

### 2.2 Data sources

The study is based on a matched cohort in which patients are matched for gender and birth year with patients without congenital heart disease. Data from the Swedish National Patient Register and Cause of Death Register were used. Swedish residents have access to publicly funded inpatient and outpatient healthcare. Individuals with UVH are generally diagnosed and followed by hospital-based cardiologists in specialized outpatient clinics. Each individual residing in Sweden is provided a unique personal identity number that allows linking different register data to each other in an unbiased manner [21]. The Swedish National Patient Register was introduced in 1964 and has had full coverage since 1987. Therefore, reporting to this register is mandatory for all hospitals. Data on outpatient specialist care have been available from the Swedish National Patient Register since 2001. The outpatient part of the Swedish National Patient Register has high (>80%) coverage of all somatic specialized outpatient visits that are provided in the public health care system [22]. Diagnoses in the Swedish National Patient Register are coded in accordance with the International Classification of Diseases (ICD). The Cause of Death Register contains information on all deaths of Swedish citizens from 1961 onwards.

### 2.3 Study population

We linked data from the Swedish National Patient Register and Cause of Death Register to identify patients with UVH who were born between 1 January 1970 and 31 December 2017. The study population in this study was patients born between 1970 and 2017, why the oldest patients in our population are 47 years old. UVH was defined as any of the following congenital heart defects: HLHS, pulmonary atresia with intact ventricular septum (PAIVS), tricuspid atresia (TA), hypoplastic right heart syndrome (HRHS), double inlet left ventricle (DILV), and double outlet right ventricle (DORV). The congenital heart defect diagnoses were coded according to the ICD, Eighth Revision (ICD-8), Ninth Revision (ICD-9), and Tenth Revision (ICD-10) (Supplemental Table 1). The diagnosis of UVH was defined as a patient having at least one registered ICD code diagnosis of a congenital heart defect categorized as UVH during at least one outpatient department visit, at hospital discharge, or on a death certificate between 1970 and 2017.

We identified 5075 patients (Supplemental Table 2) with UVH who were born during the study period and were registered in the National Hospital Inpatient Register, Outpatient Register, or National Cause of Death Register in Sweden. A single patient may have had more than one UVH diagnosis, but the diagnosis that was first registered was considered the main one. Follow-up was from the date of birth, regardless of the date of diagnosis. Data were collected until the time of death, transplantation, or until the end of the study on 31 December 2017. Approximately 10 individuals without congenital heart disease from the general population (controls) were matched for birth year and sex for each patient with UVH using the Total Population Register in Sweden. All personal identifiers were replaced by anonymized codes in the final dataset. Patients and controls were followed from January 1970 to December 2017.

The study population was divided into 4 different birth periods depending on the year of birth as follows: birth period 1, 1970–1981; birth period 2, 1982–1993; birth period 3, 1994–2005; and birth period 4, 2006–2017. Demographic data and information on medical and surgical history were retrieved from the Swedish National Patient Register.

### 2.4 Statistical analysis

Demographic data are shown as descriptive statistics to show long-term mortality in patients with UVH. With regard to descriptive statistics, continuous variables are shown as the mean with standard deviation and categorical variables as the number and percentage. The follow-up time was estimated from the date of birth until death, transplantation, or the end of the study on 31 December 2017 (whichever occurred first) in the UVH and control populations. The incidence rate is reported per 1000 person-years and was calculated as the total number of deaths divided by the total follow-up time. A Kaplan–Meier analysis was used to estimate the survival probability with the 95% confidence interval (CI) for patients with UVH and controls, separately. The Stratified Cox proportional hazards model (univariate model) was used to estimate hazard ratios (HRs) with 95% CIs to compare the UVH population with matched controls (reference group). All statistical analyses were performed with R software using packages “tidyverse” (for data management) [23] and “survival” (for Kaplan-Meier and Cox regression models) [24]. Multivariable Cox regression analysis was not performed as the patients with UVH in this study were followed up from the date of birth when few comorbidities were present.

### 2.5 Ethics

The present study was approved by the Gothenburg Regional Research Ethics Board and was performed according to the Declaration of Helsinki (Gbg 912-16, T619-18). Informed consent was waived. Social security numbers were anonymized with a unique code according to the procedures of the Swedish National Board of Health and Welfare. This study followed the recommendations of the Strengthening the Reporting of Observational Studies in Epidemiology (STROBE) Initiative.

## 3 Results

### 3.1 Baseline characteristics of the study population

We included 5075 patients with UVH and 50,620 matched controls. The median follow-up time was 13.6 (IQR 0.7; 26.8) years, and the maximum follow-up time was 47 years. A total of 758 (14.9%) patients in the UVH population were diagnosed with HLHS. The majority (95%) of patients with UVH were born in Sweden. The characteristics of the study population are shown in Table 1. Of the 1208 children born with UVH in the first time period, 470 (38.9%) died during their first year of life. In the last time period, fewer patients were born, and more patients survived. A total of 132 of 827 (15.9%) patients died during the first year of life in the last time period.

**Table 1:** Characteristics of patients with UVH and matched controls.

		UVH				Controls			
		n = 5075				n = 50,620			
Birth period		1970–1981	1982–1993	1994–2005	2006–2017	1970–1981	1982–1993	1994–2005	2006–2017
Number of patients		1208 (23.8)	1815 (35.8)	1225 (24.1)	827 (16.3)	12,080 (23.9)	18,150 (35.9)	12,190 (24.1)	8200 (16.2)
Sex									
Men		669 (55.4)	1030 (56.7)	694 (56.7)	472 (57.1)	6690 (55.4)	10,300 (56.7)	6940 (56.9)	4720 (57.6)
Women		539 (44.6)	785 (43.3)	531 (43.3)	355 (42.9)	5390 (44.6)	7850 (43.3)	5250 (43.1)	3480 (42.4)
Year of birth		1975.8 ± 3.3	1988.0 ± 3.4	1998.6 ± 3.7	2011.0 ± 3.3	1975.8 ± 3.3	1988.0 ± 3.4	1998.6 ± 3.7	2011 ± 3.3
Age at the end of follow-up (years)									
<1		470 (38.9)	548 (30.2)	251 (20.5)	132 (16.0)	69 (0.6)	54 (0.3)	31 (0.3)	409 (5.0)
1-17		164 (13.6)	164 (9.0)	458 (37.4)	695 (84.0)	37 (0.3)	30 (0.2)	5043 (41.4)	7791 (95.0)
18-39		271 (22.4)	1103 (60.8)	516 (42.1)	0 (0.0)	4417 (36.6)	18,066 (99.5)	7116 (58.4)	0 (0.0)
40-47		303 (25.1)	0 (0.0)	0 (0.0)	0 (0.0)	7557 (62.6)	0 (0.0)	0 (0.0)	0 (0.0)

Values are expressed as n (%) or mean ± standard deviation. Abbreviations: UVH = univentricular heart.

### 3.2 Mortality rate

The overall mortality rate (also including data from patients that underwent heart transplantation or heart and lung transplantation) was 37.9% (n = 1992) in the UVH cohort and 1% (n = 485) in the controls during the study period. The incidence rate of mortality per 1000 person-years was 24.1 in patients with UVH and 113.7 in patients with HLHS (Table 2, Table 3). The incidence rate of mortality was higher in patients with UVH in general and in those with HLHS than in controls in all birth periods. The highest incidence rate of mortality was found in patients with HLHS. Men with HLHS had an incidence rate of 143.9 per 1000 person-years and women had an incidence rate of 84.6 per 1000 person-years. The incidence rate in patients with HLHS for the different birth periods varied because of the small populations in these cohorts. The incidence rate of death within the first year of life declined from 660.5 per 1000 person-years born in 1970–1981 to 427.1, 268.7, and 139.0 born in 1982–1993, 1994–2005, and 2006–2017, respectively, in all patients with UVH. From 1 year and up to a maximum of 47 years, the incidence rate of death per 1000 person-years declined from 12.4 in 1970–1981 to 8.6 in 2006–2017.

**Table 2:** Incidence rate of mortality in the study population according to sex and birth.

	Total number of		IR		Number of patients		IR		Number of patients		IR	
	patients with UVH				with HLHS				with other types of UVH			
	UVH	Controls	UVH	Controls	HLHS	Controls	HLHS	Controls	Non-HLHS	Controls	Non-HLHS	Controls
All patients	5075	50,620	24.08	0.37	758	7580	113.74	0.34	4317	43,040	18.68	0.37
Men	2865	28,650	24.91	0.44	467	4670	143.87	0.37	2398	23,980	18.60	0.45
Women	2210	21,970	23.05	0.28	291	2910	84.58	0.28	1919	19,060	18.78	0.27
Birth period												
1970–1981	1208	12,080	30.34	0.47	95	950	76.52	0.34	1113	11,130	28.39	0.49
1982–1993	1815	18,150	22.56	0.31	274	2740	207.32	0.35	1541	15,410	15.99	0.30
1994–2005	1225	12,190	17.57	0.26	241	2410	86.61	0.31	984	9780	9.91	0.25
2006–2017	827	8200	27.61	0.38	148	1480	77.84	0.32	679	6720	19.84	0.39

Values are expressed the number of patients or the IR per 1000 person-years. Abbreviations: HLHS = hypoplastic left heart syndrome; IR = incidence rate; UVH = univentricular heart.

**Table 3:** Mortality risk in patients with univentricular heart and matched controls according to sex and birth.

	UVH			HLHS		Non-HLHS	
	Patients with UVH who died/total patients with UVH, n (%)	Controls who died/total controls, n (%)	HR (95% CI)	Patients with HLHS who died/total patients with HLHS, n (%)	HR (95%, CI)	Patients without HLHS who died/total patients without HLHS, n (%)	HR (95%, CI)
All patients	1922/5075 (37.9)	485/50,620 (1.0)	53.0 (48.0–58.6)	516/758 (68.1)	163.5 (124.3–215.2)	1406/4317 (32.6)	42.7 (38.3–47.6)
Men	1103/2865 (38.5)	326/28,650 (1.1)	46.32 (40.9–52.5)	321/467 (68.7)	164.3 (116.7–231.5)	782/2398 (32.6)	35.9 (31.4–41.2)
Women	819/2210 (37.1)	159/21,970 (0.7)	66.86 (56.4–79.3)	195/291 (67.0)	176.7 (110.0–283.8)	624/1919 (32.5)	56.4 (46.9–67.8)
Birth period							
1970–1981	722/1208 (59.8)	237/12,080 (2.0)	48.4 (41.7–56.1)	74/95 (77.9)	116.1 (63.8–211.1)	648/1113 (58.2)	45.2 (38.8–52.6)
1982–1993	754/1815 (41.5)	166/18,150 (0.9)	60.1 (50.7–71.1)	238/274 (86.9)	122.5 (87.9–170.8)	516/1541 (33.5)	46.4 (38.4–56.0)
1994–2005	311/1225 (25.4)	61/12,190 (0.5)	59.0 (44.8–77.7)	153/241 (63.5)	6910.2 (302.0–158130.2)	158/984 (16.1)	36.7 (26.5–50.8)
2006–2017	135/827 (16.3)	21/8200 (0.3)	69.0 (43.6–109.3)	51/148 (34.5)	208.4 (65.0–668.0)	84/679 (12.4)	48.8 (29.3–81.2)

Values are expressed as n (%) or the incidence rate per 1000 person-years. Abbreviations: CI = confidence interval; HLHS = hypoplastic left heart syndrome; HR = hazard ratio; UVH = univentricular heart.

Central illustration shows an increased survival rate in patients with UVH, especially in those with HLHS, in patients born in later birth periods (1994–2005 and 2006–2017). The Kaplan–Meier survival curve in patients with UVH diverged, mostly within the first year of life, and the probability of death in patients with UVH remained higher than that in controls during the follow-up of 47 years (Fig. 1). The trends in patients with UVH and matched controls according to the birth period are shown in Fig. 2a. Survival was markedly increased in patients with UVH who were born in the 1980s, 1990s, and 2000s. Survival probabilities divided into birth periods with a comparison of patients with HLHS and the rest of the UVH population are shown in Fig. 2b and c. The differences in mortality between patients with HLHS and the rest of the UVH population were smallest in the latest birth cohort, but still substantial. Patients with HLHS had a lower survival rate than those with other types of UVH. The survival probability increased mainly in the last birth period of 2006–2017, but it was still <75%.

**Fig. 1:**
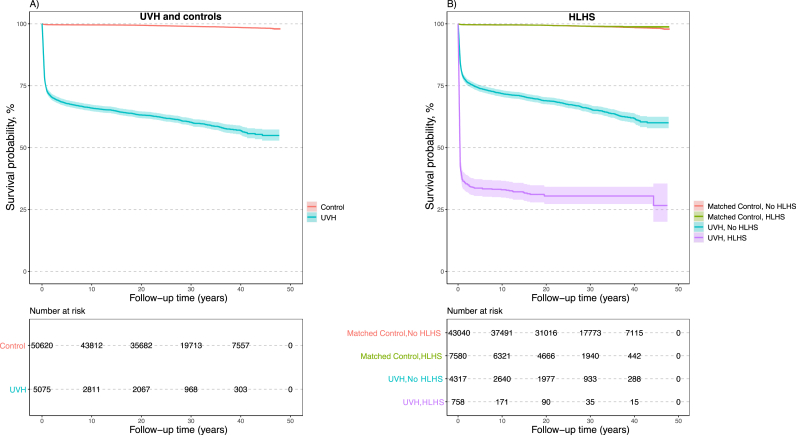
Survival curve in patients with UVH and matched controls. Survival probabilities in patients with UVH and in those with HLHS diverged mostly within the first year of life. The probability of death in patients with UVH and in those with HLHS remained higher than that in controls during the follow-up of 47 years. Abbreviations: HLHS = hypoplastic left heart syndrome; UVH = univentricular heart.

**Fig. 2:**
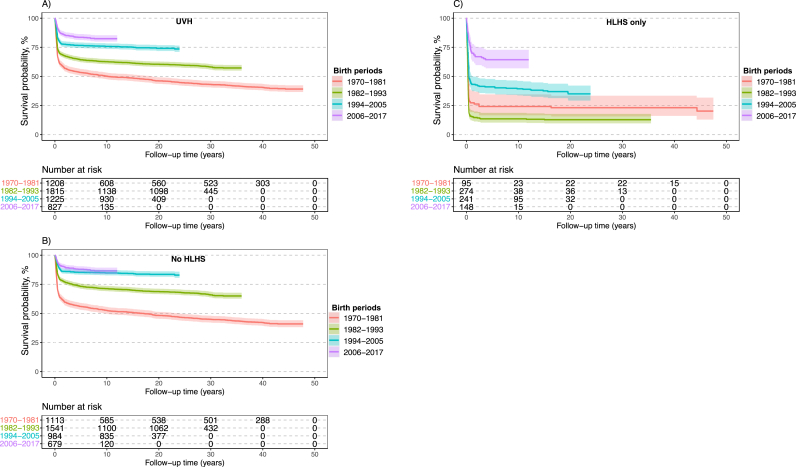
Survival curves according to the type of defect and birth periods. Survival markedly increased in patients with UVH who were born in the 1980s, 1990s, and 2000s. The differences in survival between HLHS and other types of UVH were smaller in the latest birth cohort but were still substantial. Patients with HLHS had a lower survival rate than those with other types of UVH. Abbreviations: HLHS = hypoplastic left heart syndrome; UVH = univentricular heart.

Supplemental Fig. 1a shows Kaplan–Meier curves of survival probabilities from birth in patients with UVH during long-term follow-up divided by men and women. No significant difference in the survival probability was found between men and women. Supplemental Fig. 1b shows Kaplan–Meier curves for the HLHS and non-HLHS groups, and there was no difference in the survival probability between men and women.

Supplemental Fig. 2 shows the survival probability in patients with UVH with and without surgery. Survival probability in patients who survived Fontan surgery in the first year of life was almost 90% and in non-operated patients was less than 50%.

Supplemental Fig. 3 shows outcomes based on the lesions separately. The highest survival probability is for isolated TA and the lowest survival probability is for isolated HLHS.

### 3.3 Mortality risk

The overall HR was 53.0 (95% CI, 48.0–58.6) in patients with UVH and 163.5 (95% CI, 124.3–215.2) in patients with HLHS, compared with controls (Table 3). During all birth periods, the mortality risk in patients with UVH in general and in those with HLHS was markedly higher than that in controls. The HR of mortality in the first year of life decreased from 108.2 for patients with UVH born in 1970–1981 to 53.5 in 2006–2017. However, the HR of mortality from 1 up to 47 years of age increased from 37.8 in 1970–1981 to 149.0 in 2006–2017. In the UVH population, the HR was 48.4 (95% CI, 41.7–56.1) in those born in 1970–1981, and the HR was 69.0 (95% CI, 43.6–109.3) in those born in 2006–2017. The highest risk of mortality was found in patients with HLHS (HR, 164.3; 95% CI, 116.7–231.5). Supplemental Table 3 shows the baseline characteristics of patients with HLSH and controls. The only difference between these patient groups was that there was a higher number of men in the HLHS population than in controls. In the HLHS population, 61.6% were men and 38.4% were women. The mortality rate was 68.1% in the HLHS population and 32.6% in the non-HLHS population during the follow-up period.

The incidence rate of death or transplantation in patients with UVH without HLHS, declined by 50% each decade between the 1970s and the early 2000s. From the early 2000s, the incidence rate of mortality/transplantation was stable (Supplemental Table 4). Among patients with HLHS who died or underwent transplantation during the first year of life, 230 of 274 patients were born in 1982–1993, 136 of 241 were born in 1994–2005, and 43 of 148 were born in 2006–2017. Among patients with UVH without HLHS, the numbers of deaths/transplantations in the first year of life were 401/1113 in 1970–1981, 318/1541 in 1982–1993, 116/984 in 1994–2005, and 59/679 in 2006–2017.

Number of patients with each lesion included as UVH, including patients with repaired vs. unrepaired UVH, is presented in Supplemental Table 5.

### 3.4 Sensitivity analysis

A sensitivity analysis was conducted by excluding patients with DORV because it can result in biventricular circulation. We observed relatively similar outcomes to those in the analyses above. The incidence rate of mortality per 1000 person-years was 24.1 in patients with UVH and DORV and 27.9 in patients with UVH without DORV compared with the matched controls. Therefore, the risk of mortality was 53.0 (95% CI, 48.0–58.6) times higher in patients with UVH and DORV and 61.1 (95% CI, 54.4–68.6) times higher in patients with UVH without DORV than that in controls.

A sensitivity analysis on patients with UVH that were operated on shows that 32% of deaths occurred within the first 30 postoperative days, and 58% within the first postoperative year. The corresponding IR for death were 96.04 and 0.60, respectively.

## 4 Discussion

This large nationwide cohort study showed that the overall risk of mortality, including that from transplantation, was more than 50 times higher in patients with UVH than in matched controls. Nevertheless, an increased survival rate was observed in patients with UVH over time and in later birth periods. These findings suggest that surgical and medical treatments in this vulnerable group of patients with complex congenital heart disease have improved over the last 3 decades.

We found that the overall risk of mortality was 53.0 times higher in patients with UVH in general and 163.5 times higher in patients with HLHS than in controls. Therefore, the highest relative risk of mortality was found in patients with HLHS. A possible explanation could be that HLHS patients represent technically more challenging surgery. Furthermore, patients with HLHS often have worse outcomes after Fontan surgery; narrowing of pulmonary arteries may require repeated stenting, and liver disease secondary to the Fontan procedure is a major long-term complication [25]. In addition, HLHS represents a significant proportion of patients with single-ventricle circulation that have a morphological right ventricle as a systemic ventricle. These and other factors may explain why HLHS has been reported to have the highest mortality of all congenital heart diseases. Another study showed that a diagnosis of HLHS accounts for 23% of all cardiac deaths in the first week of life among all live-born children [26]. Our findings are further supported by a study by Boldt et al. who investigated 99 fetuses in 1983–1999 [27]. They showed that neonatal mortality was high in fetuses with HLHS (87%) and those with UVH (50%). Our findings are further supported by a study from Birmingham Women's Hospital that reviewed 87 HLHS cases between 1994 and 1999 [28]. This previous study showed that the overall mortality rate in fetuses with a prenatal diagnosis of HLHS was 75% when terminated pregnancies were excluded.

We observed a marked decrease in mortality in patients with UVH during the study period. The incidence rate of death in the first year of life declined from 660.5 to 139.0 born in 1970–1981 and born in 2006–2017. Additionally, the HR of death decreased from 108.2 to 53.5 compared with controls between the same time periods. These findings are supported by a study that included hospitals from 47 US states and the District of Colombia (in 1998–2014), of 20,649 neonates with HLHS [29]. This previous study showed lower mortality (a decrease of 20%) in the groups born between 2006 and 2014 than in those born between 1998 and 2005 (25.3% vs. 20.6%). In our study, we found that the HR of death from 1 to a maximum of 47 years of age did not decline, but rather increased in the last decade of our study, from 37.8 in 1970–1981 to 149.0 in 2006–2017. This finding likely reflects a better survival in the first year of life, but a remaining higher risk of mortality in patients with UVH than in controls. The Australian and New Zealand Fontan Registry followed 1006 survivors (among 1089 patients) who underwent the Fontan procedure and included all pediatric and adult cardiac centers [30]. The ratio of surviving patients with HLHS before 1990 increased from 1% (1/173 patients treated with the Fontan procedure) to 16% (80/500 patients treated with the Fontan procedure) after 2000.

Our study indicates a decrease in the birth prevalence of children born with functional UVH, although this is outside the primary scope of our study (Table 1). This finding has been described by other researchers [31,32] and can likely be attributed to the more widespread use of fetal echocardiography, often leading to the termination of pregnancy when UVH is diagnosed. A recent review by Roeleveld et al. reported that the termination of pregnancy after diagnosing HLHS varied from 12% to 48% [31]. In a population-based study from Denmark using data from 1977 to 2009, 703 live births with UVH and 106 termination of pregnancies owing to UVH were identified [32]. There was a clear decrease in the incidence of UVH at birth and a comparable increase in the termination of pregnancy owing to UVH in the last decade of this study [32].

Unlike other studies, we found that the survival probability increased exponentially until the latest birth cohort (2006–2017). However, one-quarter of patients with HLHS did not survive to adulthood. Although survival has improved after the introduction of the Fontan procedure in the 1970s, survival in patients with HLHS has not improved further during the last 20 years [32]. Knowledge of the high mortality rate in HLHS may be a reason why the termination of pregnancy of fetuses with UVH has also increased in Sweden. In a Swedish cohort study, there was a decrease in the incidence of live birth with HLHS, an increase in the prenatal detection rate of HLHS, an increase in termination of pregnancy owing to HLHS, and an increased rate of live birth with HLHS that was surgically treated [33].

We found that the incidence rate of death or transplantation during the first year of life in patients with HLHS increased between the 1970s and the 1980s, and then declined by 50% each decade. The initial increase in the incidence rate between the 1970s and the 1980s could be explained by increased diagnostic ability and awareness. Subsequently, a decrease in the incidence of HLHS was observed, possibly due to improved antenatal detection and an increased rate of termination of pregnancy, and improved care. We also found a decrease in the overall incidence of UVH, which could probably also be explained by improved fetal diagnostics and improved care until the 2000s. The relatively stable incidence rate of mortality/transplantation after the 2000s is in line with previously published first-year-of-life mortality data in the adult congenital heart disease population in general [1].

### 4.1 Strengths and limitations of the study

The main strength of our study is the use of the Swedish National Patient Register and Cause of Death Register in Sweden. The Swedish National Patient Register has been extensively validated and has near-complete coverage of all hospital care for congenital heart disease. The Swedish healthcare system provides universal access to healthcare at no cost. This includes universal access to health care. We have virtually no loss to follow-up since participation in registries for health care providers is mandated by law. Follow-up data were collected from the Swedish National Patient Register and Cause of Death Register, which are linked with patients’ unique personal IDs. All patients with UVH were included, even non-operated patients. Therefore, our data reflect an entire nation, including changes over time.

The study has several limitations. In all registry studies based on ICD codes, there is a risk for incorrect coding and classification of congenital heart defects. Register studies without access to clinical data are further limited because they are unable to validate the diagnoses through hospital records. Nevertheless, patients with UVH are generally diagnosed and treated at specialist adult congenital heart disease centers, which might minimize misclassification. Furthermore, the use of the classification of UVH with the eighth, ninth, and tenth versions of ICD might have had a risk of inaccuracy in the conversion of UVH diagnoses. However, validation of the diagnoses in the Inpatient Register is high (85%–95%) [34,35].

In this study, UVH was defined as any of the diagnoses of HLHS, PAIVS, TA, HRHS, DILV, and DORV. By using this definition, other uncommon types of congenital heart defects that could be defined as UVH were not included (e.g., severe forms of unbalanced atrioventricular septal defect). However, our results were not significantly affected after a sensitivity analysis and after excluding patients with DORV from the UVH population.

The distinction between a morphological right or left ventricle is of importance. However, from administrative data, based on ICD-codes, it is not always completely clear which type of systemic ventricle the patients are living with, apart from patients with HLHS. To adequately determine the type of ventricle pre- and/or postoperatively, one would have to carefully review each patient's chart, data that is not available in this kind of study. We have also tried to give some insight into this by providing outcome data for HLHS vs other UVH but also outcome data for all subgroups of UVH in our Supplemental Fig. 3.

Another limitation is the lack of information on the type of surgeries performed. There is only access to administrative data regarding each operation, but no detailed information about the specific type of surgery in each case. We cannot exclude the presence of some biventricular hearts in our population because of the lack of specific ICD codes for univentricular hearts. In addition, surgical codes may not always clearly describe whether the result was a bi- or univentricular heart. We provide data on outcomes for all subgroups of UVH in our Supplemental Fig. 3. We also provide data on the time of death for patients undergoing surgery. Further, there is no access to detailed information on the cause of death.

The study cohort is matched with the total population registry of controls without congenital heart defects. No comorbidities were present at birth, thus no multivariable analysis was performed.

Finally, the Outpatient Register was started in 2000, and patients who were treated only as outpatients before 2000 could not be identified. Data from outpatient clinics and primary care were unavailable. However, children and adults with UVH are routinely treated and followed up by specialized congenital heart units and are thus likely to be registered in the Swedish National Patient Register and the Cause of Death Register in case of death.

## 5 Conclusion

The overall risk of mortality was high in patients with UVH, but exponentially decreased over time. As expected, the highest mortality risk was in patients with HLHS. Survival probability in patients who survived Fontan surgery in the first year of life was almost 90% and in non-operated patients less than 50%. However, an increased survival rate was observed in later birth periods in patients with UVH in general and HLHS in particular. These findings add important insight into the survival trends over time in patients with UVH and may serve as a foundation for understanding the natural disease course in this patient group.

The UVH population with a completed Fontan circulation will substantially increase during the coming decades because of the increased survival rate in patients with UVH. With an aging UVH population, the comorbidity burden will increase, and this will likely increase the complexity of treating this patient group.

## Any potential conflicts of interest

There is no relationship with industry that we should disclose.

## Acknowledgement of grant support

The present study was funded by the Swedish state under the ALF agreement (grant numbers 236611 and 917361); the Swedish Heart‐Lung Foundation (grant number: 20180644); and the 10.13039/501100004359Swedish Research Council (grant number: 2018-02527).

## CRediT authorship contribution statement

**Ayse-Gül Öztürk:** Writing – original draft, Visualization, Validation, Methodology, Investigation, Formal analysis, Data curation, Conceptualization. **Mikael Dellborg:** Writing – review & editing, Visualization, Validation, Supervision, Resources, Project administration, Investigation, Funding acquisition, Formal analysis, Conceptualization. **Anna Damlin:** Writing – review & editing, Visualization, Validation, Investigation, Formal analysis, Conceptualization. **Kok Wai Giang:** Writing – review & editing, Visualization, Validation, Software, Methodology, Formal analysis, Data curation, Conceptualization. **Zacharias Mandalenakis:** Writing – review & editing, Visualization, Validation, Supervision, Resources, Project administration, Investigation, Funding acquisition, Formal analysis, Conceptualization. **Peder Sörensson:** Writing – review & editing, Visualization, Validation, Supervision, Resources, Formal analysis, Conceptualization.

## Declaration of competing interest

The authors declare that they have no known competing financial interests or personal relationships that could have appeared to influence the work reported in this paper.

## Figures and Tables

**figs1:**
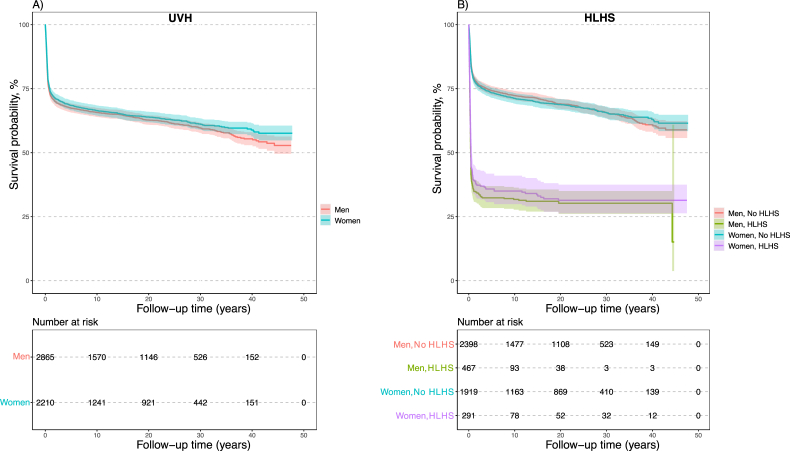


**figs2:**
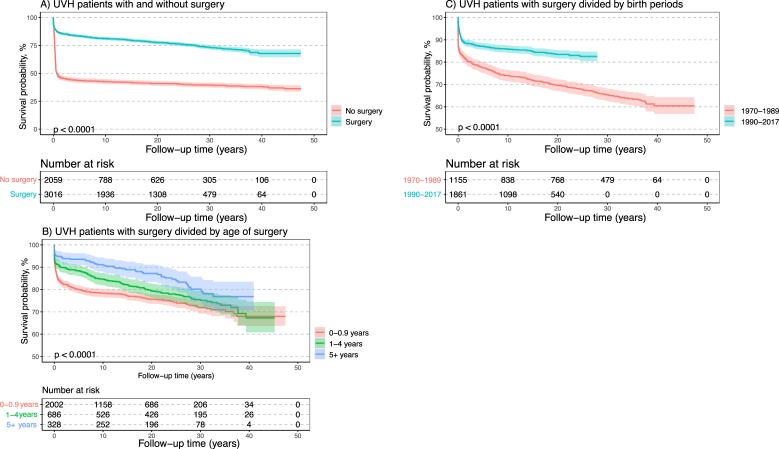


**figs3:**