# Supplementary material for: Long-term survival in patients with univentricular heart: A nationwide, register-based cohort study
Source: Int J Cardiol Congenit Heart Dis. 2024 Feb 18;15:100503. doi: 10.1016/j.ijcchd.2024.100503 (PMC11657339; doi:10.1016/j.ijcchd.2024.100503)
Supplement: Multimedia component 1 [file mmc1.docx]

**Supplemental Appendix**

Supplemental Table 1 page 2

Supplemental Table 2 page 3

Supplemental Table 3 page 4

Supplemental Table 4 page 5

Supplemental Table 5 page 6

Supplemental Figure 1 page 7

Supplemental Figure 2 page 8

Supplemental Figure 3 page 9

**Supplemental Table 1.** Diagnosis of UVH according to the ICD system.

| **UVH** | **ICD-8** | **ICD-9** | **ICD-10** |
| --- | --- | --- | --- |
| DILV | 746.37 | 745D | Q204 |
| DORV | 746.19 | 745B | Q201 |
| HLHS | 746.74 | 746H | Q234 |
| HRHS | 746.69 | 746B | Q226 |
| PAIVS | 746.64 | 746A | Q220 |
| TA | 764.54 | 746B | Q224 |

Abbreviations: DILV = double inlet left ventricle; DORV = double outlet right ventricle; HLHS = hypoplastic left heart syndrome; HRHS = hypoplastic right heart syndrome; ICD = International Classification of Diseases; PAIVS = pulmonary atresia with intact ventricular septum; TA = tricuspid atresia; UVH = univentricular heart.

**Supplemental Table 2.** Demographics of patients with UVH according to the main diagnosis.

| Diagnosis | Total population |
| --- | --- |
| UVH | 5,075 (100) |
| DILV | 861 (17.0) |
| DORV | 1,835 (36.2) |
| HLHS | 758 (14.9) |
| HRHS | 736 (14.5) |
| PAIVS | 1,995 (39.3) |
| TA | 413 (8.1) |

Values are expressed as n (%). Abbreviations: DILV = double inlet left ventricle; DORV = double outlet right ventricle; HLHS = hypoplastic left heart syndrome; HRHS = hypoplastic right heart syndrome; PAIVS = pulmonary atresia with intact ventricular septum; TA = tricuspid atresia, UVH = univentricular heart.

**Supplemental Table 3.** Characteristics of the study population according to the type of UVH defect.

|  |  | **Non-HLHS** | **Controls** | **HLHS** | **Controls** |
| --- | --- | --- | --- | --- | --- |
|  |  | (n=4,317) | (n=43,040) | (n=758) | (n=7,580) |
|  |  |  |  |  |  |
| Sex |  |  |  |  |  |
|  | Men | 2,398 (55.5) | 23,980 (55.7) | 467 (61.6) | 4,670 (61.6) |
|  | Women | 1,919 (44.5) | 19,060 (44.3) | 291 (38.4) | 2,910 (38.4) |
| Birth year |  |  |  |  |  |
|  |  | 1,990.8±12.3 | 1,990.8±12.2 | 1,994.8±11.2 | 1,994.8±11.2 |
| Age (years) |  |  |  |  |  |
|  | <1 | 918 (21.3) | 448 (1.0) | 483 (63.7) | 115 (1.5) |
|  | 1-17 | 1,305 (30.2) | 10,442 (24.3) | 176 (23.2) | 2,459 (32.4) |
|  | 18-39 | 1,806 (41.8) | 25,035 (58.2) | 84 (11.1) | 4,564 (60.2) |
|  | 40-47 | 288 (6.7) | 7,115 (16.5) | 15 (2.0) | 442 (5.8) |
| Birth period |  |  |  |  |  |
|  | 1970-1981 | 1,113 (25.8) | 11,130 (25.9) | 95 (12.5) | 950 (12.5) |
|  | 1982-1993 | 1,541 (35.7) | 15,410 (35.8) | 274 (36.1) | 2,740 (36.1) |
|  | 1994-2005 | 984 (22.8) | 9,780 (22.7) | 241 (31.8) | 2,410 (31.8) |
|  | 2006-2017 | 679 (15.7) | 6,720 (15.6) | 148 (19.5) | 1,480 (19.5) |
| Place of birth |  |  |  |  |  |
|  | Sweden | 4,078 (94.5) | 32,601 (75.7) | 744 (98.2) | 5,916 (78.0) |
|  | Other countries | 239 (5.5) | 10,439 (24.3) | 14 (1.8) | 1,664 (22.0) |
| Mortality during follow-up | |  |  |  |  |
|  |  | 1,406 (32.6) | 427 (1.0) | 516 (68.1) | 58 (0.8) |
| Mean follow-up time | |  |  |  |  |
|  |  | 17.4±14.1 | 26.7±12.3 | 6.0±10.3 | 22.8±11.3 |

Values are expressed as n (%) or mean ±standard deviation. Abbreviations: HLHS = hypoplastic left heart syndrome; UVH = univentricular heart.

**Supplemental Table 4.** Incidence rate of mortality and transplantation during the first years according to the birth cohort.

| Birth cohort | UVH |  |  | HLHS |  |  |
| --- | --- | --- | --- | --- | --- | --- |
|  | population, n | Events,  n (%) | IR | population, n | Events,  n (%) | IR |
| 0-0.9 years |  |  |  |  |  |  |
| 1970-1981 | 1,113 | 401 (36.0) | 471.25 | 95 | 69 (72.6) | 1,502.86 |
| 1982-1993 | 1,541 | 318 (20.6) | 237.06 | 274 | 230 (83.9) | 2,175.63 |
| 1994-2005 | 984 | 116 (11.8) | 126.30 | 241 | 136 (56.4) | 925.76 |
| 2006-2017 | 679 | 59 (8.7) | 92.30 | 148 | 43 (29.1) | 351.61 |
|  |  |  |  |  |  |  |
| 2-4.9 years |  |  |  |  |  |  |
| 1970-1981 | 662 | 39 (5.9) | 20.29 | 25 | 2 (8.0) | 28.57 |
| 1982-1993 | 1,186 | 56 (4.7) | 16.14 | 41 | 3 (7.3) | 25.96 |
| 1994-2005 | 847 | 10 (1.2) | 3.96 | 102 | 3 (2.9) | 10.07 |
| 2006-2017 | 548 | 10 (1.8) | 6.82 | 80 | 6 (7.5) | 29.59 |

Values are expressed as n, n (%), or the IR. Abbreviations: HLHS = hypoplastic left heart syndrome; IR = incidence rate; UVH = univentricular heart.

**Supplemental Table 5**. Numbers of patients with each lesion included as UVH, including patients with repaired vs. unrepaired UVH.

|  | N (%) | Repaired | Unrepaired |
| --- | --- | --- | --- |
| DILV | 384 (7.6) | 107 | 277 |
| DORV | 1255 (24.7) | 879 | 376 |
| HLHS | 758 (14.9) | 266 | 492 |
| HRHS | 139 (2.7) | 90 | 49 |
| Non-isolated UVH | 1235 (24.3) | 865 | 370 |
| PAIVS | 1202 (23.7) | 733 | 469 |
| TA | 102 (2.0) | 76 | 26 |

Values are expressed as n (%). Abbreviations: DILV = double inlet left ventricle; DORV = double outlet right ventricle; HLHS = hypoplastic left heart syndrome; HRHS = hypoplastic right heart syndrome; PAIVS = pulmonary atresia with intact ventricular septum; TA = tricuspid atresia, UVH = univentricular heart.

**Supplemental Figure 1**. Survival curves in patients with UVH and HLHS divided by men and women. There were no differences in survival probability between men and women.

**
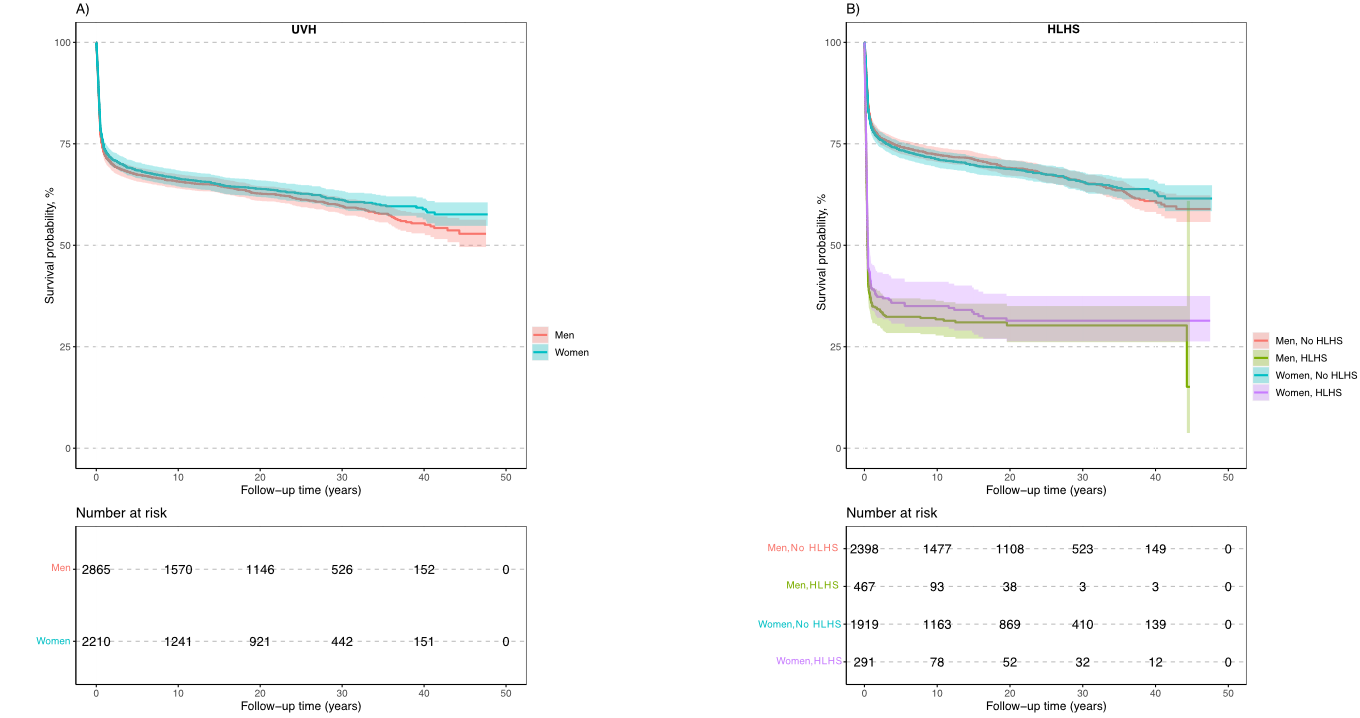
**

Abbreviations: HLHS = hypoplastic left heart syndrome; UVH = univentricular heart.

**Supplemental Figure 2.** Survival probability in patients with UVH with and without surgery. Out of 5,075 patients, 2,059 (40.6%) patients were surgically treated. Survival probability in patients who survived Fontan surgery first year of life was almost 90% and in non-operated patients less than 50%.


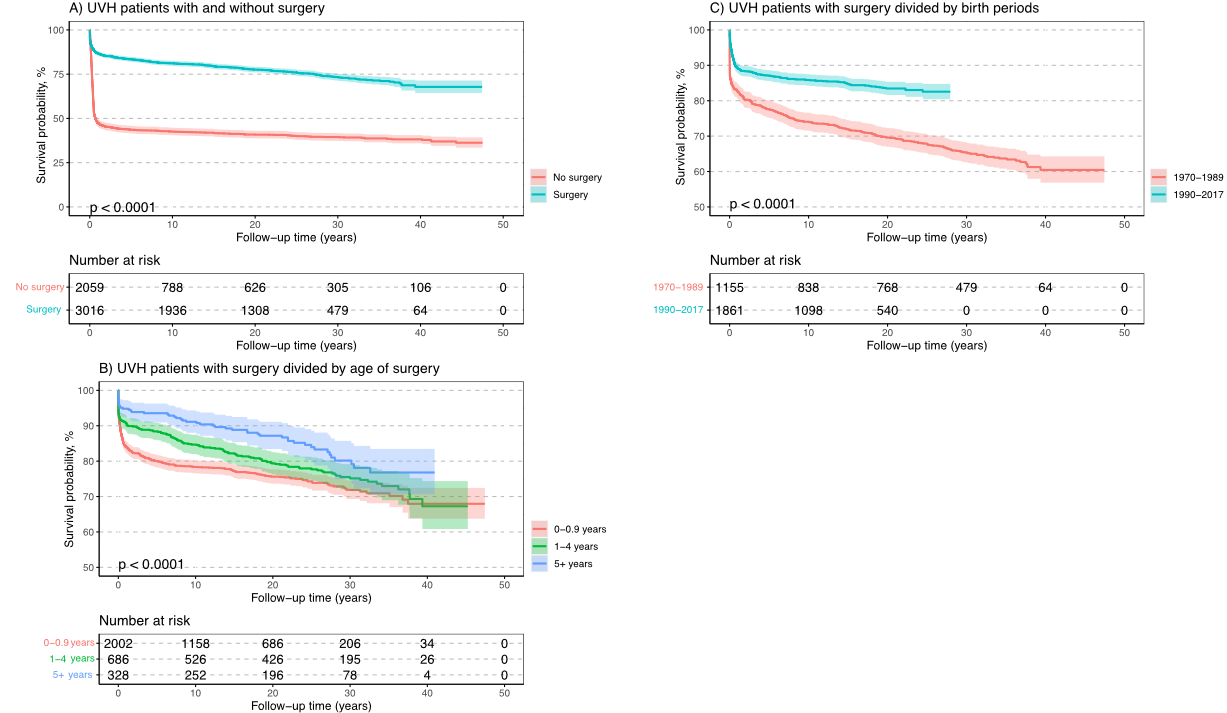


Abbreviations: UVH = univentricular heart.

**Supplemental Figure 3.** Survival outcomes based on the lesions included in UVH.

The highest survival probability is for isolated TA and lowest survival probability is for isolated HLHS.


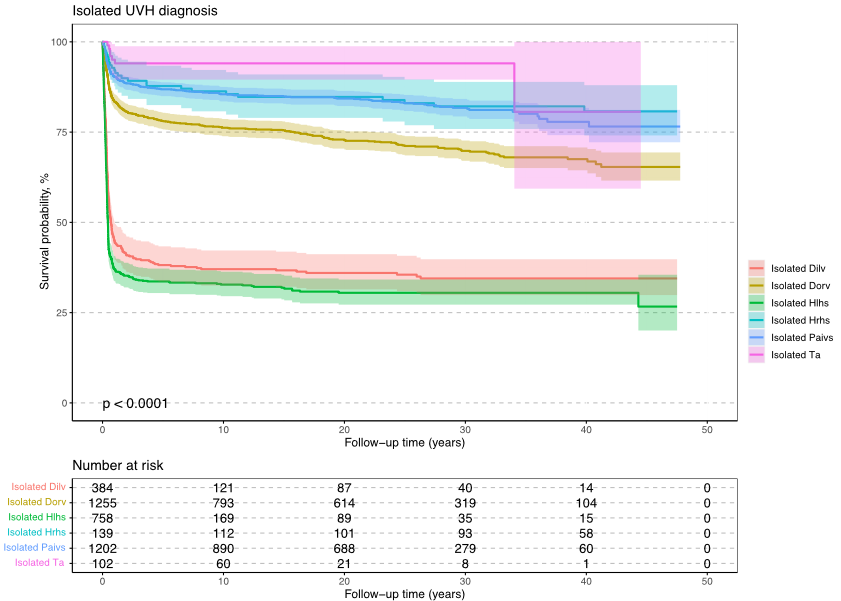


Abbreviations: DILV = double inlet left ventricle; DORV = double outlet right ventricle; HLHS = hypoplastic left heart syndrome; HRHS = hypoplastic right heart syndrome; PAIVS = pulmonary atresia with intact ventricular septum; TA = tricuspid atresia, UVH = univentricular heart.
